# The cost-effectiveness of incentive-based active case finding for tuberculosis (TB) control in the private sector Karachi, Pakistan

**DOI:** 10.1186/s12913-019-4444-z

**Published:** 2019-10-12

**Authors:** Hamidah Hussain, Amani Thomas Mori, Aamir J. Khan, Saira Khowaja, Jacob Creswel, Thorkild Tylleskar, Bjarne Robberstad

**Affiliations:** 1 Interactive Research and Development, Global, Singapore, Singapore; 2 Centre for International Health, Bergen, Norway; 3 Department of Global Public Health and Primary Care, |University of Bergen, Bergen, Norway; 4 Stop TB Partnership, Geneva, Switzerland; 5 Section for Ethics and Health Economics, Bergen, Norway

**Keywords:** Tuberculosis, Active case finding, Cost effectiveness

## Abstract

**Background:**

In Asia, over 50% of patients with symptoms of tuberculosis (TB) access health care from private providers**.** These patients are usually not notified to the National TB Control Programs, which contributes to low notification rates in many countries.

**Methods:**

From January 1, 2011 to December 31, 2012, Karachi’s Indus Hospital - a private sector partner to the National TB Programme - engaged 80 private family clinics in its catchment area in active case finding using health worker incentives to increase notification of TB disease. The costs incurred were estimated from the perspective of patients, health facility and the program providing TB services. A Markov decision tree model was developed to calculate the cost-effectiveness of the active case finding as compared to case detection through the routine passive TB centers. Pakistan has a large private health sector, which can be mobilized for TB screening using an incentivized active case finding strategy. Currently, TB screening is largely performed in specialist public TB centers through passive case finding. Active and passive case finding strategies are assumed to operate independently from each other.

**Results:**

The incentive-based active case finding program costed USD 223 per patient treated. In contrast, the center based non-incentive arm was 23.4% cheaper, costing USD 171 per patient treated. Cost-effectiveness analysis showed that the incentive-based active case finding program was more effective and less expensive per DALY averted when compared to the baseline passive case finding as it averts an additional 0.01966 DALYs and saved 15.74 US$ per patient treated.

**Conclusion:**

Both screening strategies appear to be cost-effective in an urban Pakistan context. Incentive driven active case findings of TB in the private sector costs less and averts more DALYs per health seeker than passive case finding, when both alternatives are compared to a common baseline situation of no screening.

**Electronic supplementary material:**

The online version of this article (10.1186/s12913-019-4444-z) contains supplementary material, which is available to authorized users.

## Background

In Asia efforts to strengthen tuberculosis (TB) care are concentrated in the public sector even though over 50% of patients with symptoms of TB access health care from private sector [1–3]. Private general practitioners (GPs) are often preferred as they are easily accessible and offer flexible hours of service compared to public sector facilities [4]. These individuals, if diagnosed with TB, are often not notified to the National TB Control Programs (NTPs), and therefore reduces notification rates in many countries. To identify TB patients being treated in the private sector, public programs have worked with private providers utilizing strategies such as active case finding and incentives for health workers [5–8].

The effectiveness of involving the private sector to identify people with TB has been demonstrated [9, 10], though with limited data on its cost-effectiveness [11, 12]. TB programs have used incentives to keep the private sector engaged for improved case finding, expand access to treatment and to improve outcomes. However, there has been diverging results with some studies showing benefits from incentives and others no benefits [13–15]. In addition, very limited information is available on cost-effectiveness of the incentives to the health care providers to increase case notification as part of active case finding programs.

Pakistan ranks fifth amongst TB high-burden countries worldwide. It accounts for 61% of the TB burden in the WHO Eastern Mediterranean Region, yet only 58% of the estimated people with TB are detected and notified through the standard passive system [16]. It is speculated that many of these missing TB cases are treated in the private sector. Indus Hospital, a private sector hospital based in Karachi, engaged private providers and provided incentives to health worker in its catchment area to increase TB case detection [17]. All TB patients were notified to the NTP, and received standard of care from their neighborhood GP provider. This study estimated the cost- effectiveness of this active case finding program in the private sector using incentives as compared to the existing passive case finding and treatment program.

## Methods

### Setting

In Pakistan general health care services are delivered by two parallel, independent and competing public and private health systems. The public sector provides free consultation at outpatient level but patients have to pay for their medications, whereas, the private sector operates as a fee for service system. An estimated 67.4% households in Pakistan consult private health providers when they need medical services with the province of Sindh leading at 78.93% [18].

The study was conducted in Korangi Town**,** one of 18 towns of Karachi, Sindh, and consisted of both active case finding (ACF) and passive case finding (PCF). The PCF was based at the Indus Hospital, which is a private not for profit tertiary care hospital that provides free quality health care services to patients. The hospital is primarily funded through donations and serves an estimated population of 800,000 people from all five major ethnic groups of Pakistan, mostly lower-income households. The TB clinic at the Indus Hospital is part of the standard TB treatment and reporting system in Pakistan. It provides care to 150–200 walk-in patients daily who presents themselves with symptoms of TB. Patients are investigated with smear microscopy, Xpert MTB/RIF assay and chest x-rays. If diagnosed, they are treated and reported based on the national TB program guidelines.

ACF was conducted for 2 years from January 1, 2011 to December 31, 2012. Indus Hospital’s TB outreach program created a network of over 80 stand-alone private family clinics in its catchment population to increase TB case detection and treatment for TB. The family clinics were selected based on their geographical dispersion, high patient load and proximity to high-risk population.

The active case finding included monetary and non-monetary incentives to both health workers and the family doctors providing the treatment. A project-employed health worker was stationed at each of the family clinics to verbal symptom screen all patients and their attendants seeking health care for any illness and refer people with potential TB symptoms for further evaluation by the family physician which included smear microscopy, Xpert MTB/RIF assay and chest x-rays. The health workers received a fixed monthly stipend and performance based incentive payment for submitting a daily phone report ($0.18 per report), procuring an acceptable sputum sample ($0.88), and identifying a smear-positive case ($11.80) or other form of tuberculosis ($5.88). Additional incentives were provided for treatment initiation ($1.76) and each follow-up visits ($0.58).

The family clinic doctors received non-monetary incentives such as communication material, height charts, weighing scales and advertisement on local cable channels that increased the publicity of the clinic. The program also conducted a media campaign using billboards, banners, pamphlets, posters, and short advertisements on local cable television channels. The message focused upon the need for anyone with over 2 weeks of productive cough to seek free testing and free treatment at either the Indus hospital or associated family clinics. All patients were provided free diagnostics and treatment as per the national TB program guidelines. Individuals enrolled in both arms were assigned unique identifiers. A total of 129 participants who were included in the costing study were randomly selected from 859 patients in passive arm and 1858 patients in the active arm that had been on treatment for a minimum of 2 months during the same time frame. Out of these 129 patients who were interviewed for out of pocket expenditure they incur in the process of seeking TB care, 45 received treatment at the Indus hospital and the remaining 84 at the private family clinics. There was no cross over of patients between the 2 arms.

### Notification data

Data were collected on all people with presumptive TB, smear microscopy conducted, people diagnosed with TB and treatments initiated. Treatment outcomes (cure, loss to follow up and death) were collected from the routine reporting system of the NTP. On average loss to follow up occurred at month 3 and death at month 4 for this cohort. As we did not have outcome information on patients transferred out to other treatment centers, they were considered as loss to follow up by our study [19].

### Cost data

We collected data on the direct costs from the perspective of the patients, health facilities and the TB program, as well as indirect costs from the patients’ perspective. The patient cost collection tool used in this study was adapted from a World Bank questionnaire on economic impact of adult fatal illness [20]. This tool was adapted for TB and has been used in multiple TB cost studies in Canada, Haiti and Dominican Republic [21–24]. The questionnaire was translated into Urdu and pilot-tested. We recruited an experienced team of field workers and trained them in interview ethics, techniques and procedures. After verbal informed consent, interviews were conducted at home after treatment initiation. The data capture was electronic on smart phones using an open-source Open Data Kit (ODK) platform. Quality control procedures were put in place through regular field supervision of interviewers and daily review of collected data.

The patient’s costs were estimated at three points of time, including when they sought care for the TB symptoms (pre-diagnosis), at diagnostic visits and during the treatment phase. The cost categories included outpatient visits, physician consultation, hospitalization, laboratory tests, radiology and drugs. Cost per visit to a health care provider were calculated and extrapolated for the entire duration of treatment. Indirect costs were estimated from time spent to access care including travel and hours spent at the health facility. Time was then converted to a monetary value based on average wages earned prior to the diagnosis of TB as reported by the patients.

Health facility costs included outpatient clinics, hospitalization and laboratory services. Existing hospital and program accounting systems were reviewed and the availability of cost and other data assessed. For all activities related to the treatment of tuberculosis health facility and program resources were quantified and organized by categories including personnel, incentives, diagnostic test, monitoring and communication. The program personnel time costs were estimated using activity based costing (ABC). Time spent on activities related to patient care was determined by interviews and following personnel during their routine actives. Personnel salaries were then allocated based on the time spent for care of TB patients.

Costs were collected in Pakistan Rupees 2012 and converted to US dollars using the average exchange rate for the year by State Bank of Pakistan (1 USD = 93.29 PKR). Cost data were analyzed using Stata (Stata Corp LP. College Station, Texas). Costs were discounted at 3% as recommended in the literature [25].

### Decision and Markov model

**Fig. 1:**
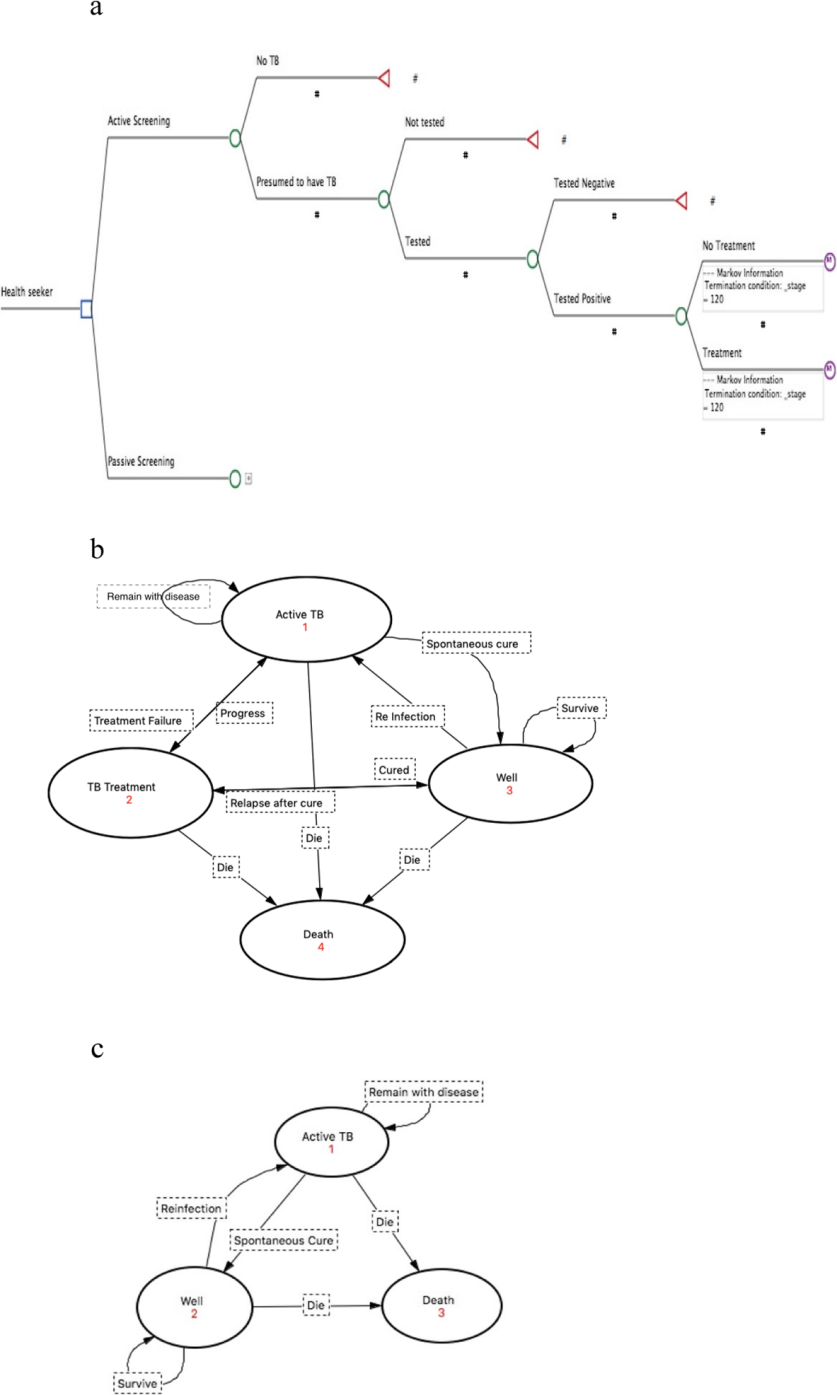
**a** Decision Tree of the model capturing the short-term outcomes of screening and testing. **b** State Transition Diagram illustrating the Markov model capturing longer-term costs and health effects when TB patients receive treatment. **c** State Transition Diagram illustrating the Markov model capturing longer-term costs and health effects for TB patients in the absence of treatment

The cost-effectiveness analysis compared costs and health outcomes associated with all forms of TB diagnosed and treated either through active case finding or passively at a TB center. A decision tree was created with short-term outcomes. In this model, patients in the active or passive case finding arm were identified as presumptive TB patients. People without TB symptoms were not followed further. People with symptoms were asked to test for TB. Patients found not to have TB were not evaluated further in the model. Those diagnosed with the disease were offered treatment (Fig. 1a).

The long-term outcomes of TB treatment for both intervention arms were estimated by a Markov model with four mutually exclusive health states; 1) TB treatment, 2) well, 3) active TB and 4) death. Figure 1b represents the possible movements of patient between the health states. In the model, patients started on “TB treatment” either survived or died from TB. Surviving patients completed treatment, or defaulted to return to active TB. Those that completed treatment achieved cure and entered well state, or failed treatment and returned to active TB. People in the “well state” survived or died of natural causes. Those who survived either remained well or acquired reinfection and entered TB treatment cohort [26]. People in the “active TB” state survived or died of disease. Those who survived either achieved spontaneous cure and entered well state, or returned to TB treatment. “Death” is an absorbing health state, which occurred as disease outcome or as background mortality.

The long-term outcomes for TB positive patients not receiving treatment in both intervention arms were estimated by a Markov model with three mutually exclusive health states; 1) Active TB, 2) Well and 3) Death. The health states and possible transitions are illustrated in Fig. 1c.

At the beginning of the first-year, health seekers in the TB treatment arm are assumed to start in the TB treatment state, and with a mean age of 32 years (as calculated from study data). The model was run for 40 cycles, each with a 6-month duration. Costs and outcomes were calculated and cumulated after each cycle.

### Transition probabilities

Most transition probabilities used in the model were estimated from an actual cohort of patients seen during this project (Table 1 and Additional file 1: Table S1). Probabilities for spontaneous resolution and relapse after cure were estimated from published cohort studies [21, 28–30]. Background mortality for the “Well” state was estimated using a WHO life table for Pakistan [32], whereas, TB specific mortality data generated from the project was used for the “TB treatment” state [17]. Comorbidities such as HIV were not considered in the model, as the prevalence of HIV is less than 1% in Pakistan. Multi drug resistant TB was not considered as the project’s objective was to increase case detection for susceptible TB.

**Table 1:** Modeling inputs, assumptions and ranges of TB outcome for health states

Probability	Active Case Finding arm (lower and upper limit)	Passive Case Finding arm (lower and upper limit)	Reference
Probability of TB Presumptive	0.022 (0.02–0.03)	0.046 (0.04–0.06)	[17, 34]
Probability of TB Test	0.61 (0.49–0.73)	0.82 (0.66–0.98)	[17, 34]
Probability of Positive TB test	0.08 (0.06–0.10)	0.13 (0.10–0.16)	[17, 34]
Probability of starting TB treatment	0.94 (0.75–1.13)	0.94 (0.75–1.13)	[17, 34]
Transition Probabilities			
Probability of Successful TB treatment	0.77 (0.62–0.92)	0.75 (0.60–0.90)	[17, 34]
Probability of Failing TB treatment	0.02 (0.01–0.02)	0.04 (0.03–0.05)	[17, 34]
Probability of default during TB treatment	0.19 (0.15–0.23)	0.20 (0.16–0.24)	[17, 34]
Probability of Death during TB treatment	0.02 (0.01–0.02)	0.01 (0.008–0.012)	[17, 34]
Probability of dying from natural causes	Life tables	Life tables	[26]
Probability of relapse after successful treatment	0.03 (0.02–0.04)	0.03 (0.02–0.04)	[28]
Probability of mortality if no TB treatment	0.33 (0.26–040)	0.33 (0.26–0.40)	[29]
Probability of spontaneous cure	0.25 (0.20–0.30)	0.25 (0.20–0.30)	[30]
Cost			
Cost of no TB symptom at screening	$2.15 (1.72–2.58)	$4.24 (3.39–5.09)	Additional file 1: Table S1
Cost if no TB test with positive symptom	$2.15 (1.72–2.58)	$4.24 (3.39–5.09)	Additional file 1: Table S1
Cost of screening and negative sputum test	$7.15 (5.72–8.58)	$11.24 (8.99–13.49)	Additional file 1: Table S1
Cost of screening and no treatment if smear positive	$82 (65.60–98.40)	$48 (38.40–57.60)	Additional file 1: Table S1
Cost of screening, tests and successful TB treatment	$223 (178.40–267.60)	$171 (136.80–205.20)	Table 2
Cost of screening, testing and TB treatment before loss to follow-up	$112 (89.60–134.40)	$86 (68.80–103.20)	Additional file 1: Table S1 (loss to follow up at 3 month of TX)
Cost of screening, testing and TB treatment before death from disease	$149 (119.20–178.80)	$114 (91.20–136.20)	Additional file 1: Table S1 (Death at 4 month of TX)
Disease weights (DWs)			
DW for no TB treatment	0.0.331 (0.26–0.40)	0.0.331 (0.26–0.40)	[31]
DW of well state	0	0	
DW of death	1	1	

### Health outcome

Treatment outcomes were reported by the study. Health benefits were estimated as disability-adjusted life years (DALYs), based on disease weights for TB without HIV using assumption from the Global Burden of Disease 2010 study [31] and were discounted at 3%.

### Cost-effectiveness

As discussed above public and private sector in Pakistan are parallel systems. Therefore, we treat both public and private systems as independent interventions, and consequently compared both strategies to a common baseline of “no case finding” (patients found passively in the public sector) [33]. The comparator “no case finding” represents a situation where TB patients remain unidentified and consequently experience the natural path of the disease. While this is arguably an artificial situation, this analytical approach enables the comparison of these independent interventions as well as comparison between TB screening and health interventions in other parts of the health services more broadly. The results are presented as incremental costs, incremental benefits and incremental cost-effectiveness ratios (ICERs) compared to no case finding, expressed as cost (2012 USD) per DALY averted.

### Uncertainty and sensitivity analyses

Sensitivity analyses were conducted to explore the effects of key parameters. Upper and lower values (+/− 20%) for each parameter were estimated around mean value and standard error (SE) was calculated by (upper-lower)/ (1.96*2) [25]. The model was designed by using TreeAge Pro 2015 (TreeAge Pro Inc., Williamston, MA).

Ethical approval for the study was obtained from Interactive Research and Development IRB.

IRB - IRD is registered with the U.S. Department of Health and Human Services (DHHS) Office for Human Research Protections (OHRP) with registration number IRB 00005148.

## Results

Probabilities of health seekers screened, tested, treatment initiated and treatment outcome were obtained from the larger study over a period of 2 years and reported for the first year of study elsewhere (Table 1) [17, 34].

Of the 129 patients interviewed, males and females were equally represented and most patients were between 15 and 44 years of age. The average household size was 6.5 people. Over 50% of the patients had received at least primary education. Only 30% of patients were employed at the time of interview and 8% of these experienced a decrease in income. In the ACF arm 52% had smear negative TB whereas, 42% had smear negative TB in the PCF arm.

The incentive-based ACF program incurred $223 per patient treated (program: $164, patient: $59) whereas $171 per patient (program: $100, patient: $71) was spent at the PCF non-incentive arm. ACF resulted in 999 more patients diagnosed and treated for TB as compared to the passive arm at the cost of USD 272 per additional patient. On average, it took at least 5 symptomatic visits to a health facility at a cost of $4 per visit before smear microscopy was requested in the passive arm as compared to 2 visits at $2 per visit in the active case finding arm. In the PCF arm, almost 50% of the direct out-of-pocket expenditure by patients was pre-diagnosis of TB. Combined the main cost drivers were non-TB medication (51%) and food and transport (33%). Indirect costs for patients treated at the passive center were higher than those treated at the private family clinics. In the active case finding program substantial costs were spent on personnel and incentives. A USD 40 per patient incentive was provided to the health workers for case detection and case holding. In the PCF arm, personnel cost was the major cost driver (Table 2).

**Table 2:** Provider and patient costs for TB screening, diagnosis and treatment

Provider costs	Average cost per patient (USD)	
	Active case finding arm	Passive case finding arm
	(*N* = 1858)	(*N* = 859)
Personnel	59 (36%)	44 (44%)
Incentive	40 (24%)	0 (0%)
Diagnostic Test	11 (7%)	11(11%)
Anti TB Drugs	22 (13%)	22 (22%)
Supervision and Monitoring	18 (11%)	9 (9%)
Marketing and communication	4 (2%)	6 (6%)
Training	0 (0%)	0 (0%)
Equipment	8 (5%)	8 (8%)
Mobile Xray Unit	2 (1%)	0 (0%)
Total provider cost per patient	$164	$100
Patient – out of pocket expenditure	Average cost per patient (USD)	
	Active case finding	Passive case finding
	(*N* = 84)	(*N* = 45)
Before diagnosis		
No. of visits	2	5
Consultation	0.36 (1%)	3.45 (9%)
Test	1.6 (5%)	2.48 (6%)
Medication	4.62 (15%)	10.44 (26%)
Food and Transport	0.36 (1%)	3.19 (8%)
Diagnosis		
Consultation	0.42 (1%)	0 (0%)
Test	1.55 (5%)	0.52 (1%)
Medication	12.72 (41%)	1.33 (3%)
Food and Transport	2.27 (7%)	3.03 (8%)
Treatment and follow up		
Consultation	0.69 (2%)	0 (0%)
Test	0.6 (2%)	0 (0%)
Medication	3.2 (10%)	4.21 (11%)
Food and Transport	2.79 (9%)	11.73 (29%)
*Subtotal direct cost*	$31	$40
*Lost earnings*	$28	$31
*Total cost per patient*	$59	$71
*Total program cost per patient*	$223	$171

The project did not follow patients who were not categorized as presumptive TB patients, nor those who did not provide specimen for sputum smear microscopy or tested negative for TB. Costs associated with screening these patients were estimated based on the total number of people screened in each arm [34].

### Cost-effectiveness

PCF when compared to no screening program costed 46.27 dollars per DALY saved. Whereas, ACF incrementally averted 0.1699 DALYs at the cost of 15.74 over 6 months of treatment with TB drugs when compared to baseline (Table 3).

**Table 3:** Absolute and incremental costs, effect and cost-effectiveness from the patients, health facilities and the TB program perspective. For both strategies, incremental values are calculated compared to a common baseline, i.e. no case finding

Strategy	Cost/health seeker for symptoms of TB (USD)	Incremental Cost	DALYs	Incremental DALYs	Incremental C/E
No passive case finding	0		0.1806		
Passive case finding	6.09	6.09	0.0491	0.1315	46.27
No active case finding	0		0.1806		
Active case finding	2.67	2.67	0.0107	0.1699	15.74

The DALY is a negative outcome measure, meaning that less are better than more. Thus, the DALYs were inverted for computational reasons in this analysis

### Sensitivity analysis

**Fig. 2:**
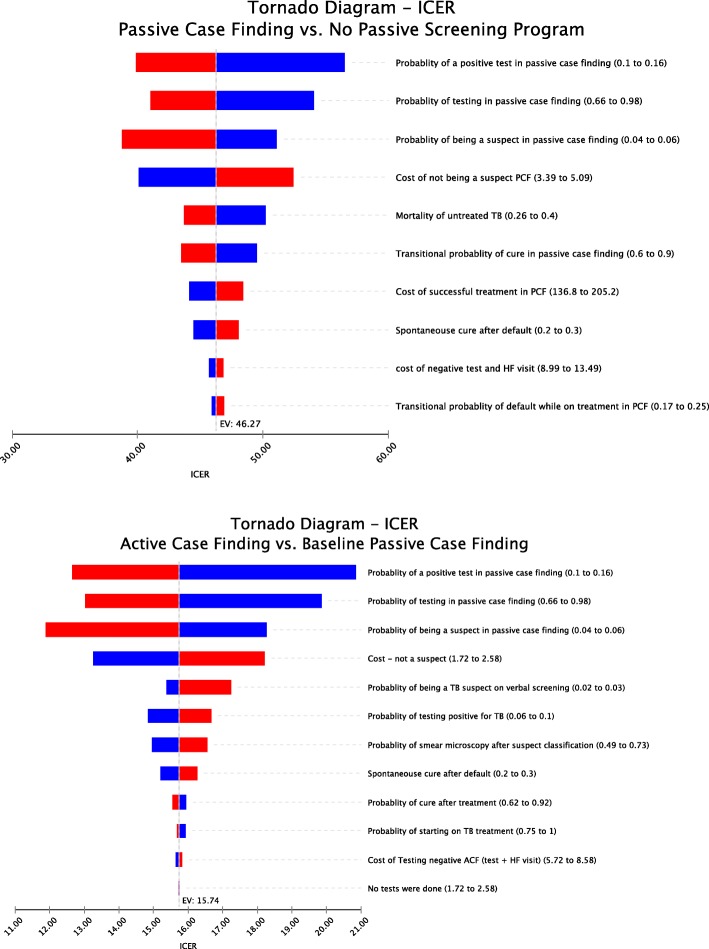
Tornado ICER diagram of active case finding as compared to passive case

A deterministic sensitivity analysis was performed for each variable in the model where the parameters and cost were varied over a predefined range (Table 1). In the Tornado diagrams for active and passive case findings variables are sorted according to their individual potential impact on the ICER (Fig. 2). The ICER in both arms was most sensitive to probability of being tested in the PCF arm and if the test was positive, but the uncertainty range for no individual variable was wide enough to change the main result that ACF appear to be more cost-effective than PCF. The uncertainty ranges in the individual cost variables had relatively little impact on cost-effectiveness.

## Discussion

Our results indicate that incentive-based ACF in the private sector is more cost-effective than PCF, and should be considered if resources permit. Also PCF is highly cost-effective when compared to a commonly applied threshold of GDP per DALY averted [27]. We believe PCF and ACF most appropriately should be considered independent in the context of public and private health sectors in Pakistan, respectively. Our analysis therefore should not interpreted in favor of disinvestments of the PCF scheme.

Even though the ACF model was over all more expensive per patient treated; it had less out-of-pocket expenditure with fewer health facility visits and better outcomes for patients than those treated in the standard passive system. People incurred substantial direct and indirect costs to access care for TB in settings where programs are otherwise said to be providing free TB services [35–37]. A systematic review reports a mean total patient cost ranging from $55 to $8198 with 20% direct expenditure, 20% indirect medical expenditure and 60% income loss, with over 50% of the costs expected before TB treatment was initiated [38]. This is very consistent with our findings that patients in Karachi experienced half the cost prior to diagnosis and over 40% attributable to income loss. In line with WHO End TB Strategy to ‘Zero TB-affected families facing catastrophic costs’ [39], policies should focus on ensuring that people with TB are identified early and are supported to minimize out of pocket expenditure. The ACF strategy used in this study doubled TB case notification rates [17], took health care closer to people and lead to earlier diagnosis at a reduced cost to the patient.

Evidence suggests that carefully planned and monitored incentives strategies can increase case detection and improve treatment completion rates and should be considered to achieve TB control goals [15]. The incentives in this program were divided over the care cycle between screening, diagnosis and treatment adherence. In addition, a strong monitoring process was put in place to improve data quality. Our analysis suggests that even with the high cost of incentive the ACF is still more cost-effective. We therefore argue that in countries where a substantial proportion of patients preferentially seek care in the private sector, scaling up incentive programs for screeners can be a cost-effective option to increase case notifications.

Our results are consistent with results from other countries such as Uganda and Cambodia that have found active case finding interventions to be cost-effective [11, 12]. The analysis suggests that active case finding is an effective strategy and should be instituted at health facilities in Pakistan whenever resources are available. This ACF program was conducted over a period of 2 years in a targeted setting in Karachi. A modelling study in the same setting has demonstrated that if the active case finding were to be sustained over a 5-year time horizon it could decrease TB mortality by over 50% and incidence of TB by 24% [34]. Whereas, models in China, South Africa and India have demonstrated that ACF will remain cost-effective even if the cost per case is USD 1000 [40]. Essentially, PCF provides certain level of coverage in TB programs and then ACF is needed to reach more people and to reach them earlier.

The strength of this analysis is that it draws on actual cost and probability data collected as part of the study. However, there are limitations; firstly, the out of pocket expenditure costs is probably an underestimate and it is unsure how the ICER might have been affected. We calculated the indirect cost based on the travel time and time spent at the health facility only for the person seeking care. In our setting, this person is often accompanied by a family members thus increasing the costs borne by the family. Second, we also could not get clear estimates for time taken off from work during illness and had to exclude this from indirect cost estimation. WHO is testing a standardized Tuberculosis Patient Cost Surveys tools in several countries which will help in estimating such costs. Third, we considered both patient and provider costs, but from the provider’s perspective we only considered operational cost and did not take capital expenditure into account for either arm of the study. Our findings may not be non-generalizable to countries where ACF and PCF strategies are undertaken by the same sector simultaneously. Studies have shown that ACF can identify people with TB earlier and therefore potentially reducing TB transmission [12, 34]. Our static model is not well suited to include effects of TB transmission, but our conclusion that ACF is cost-effective can be argued to be conservative since incorporation of reduced transmission would have made it even more favorable. It is also possible that the use of standard disease weight for TB from the Global Burden of Disease in this model overestimate the disease burden in the active arm. Especially if the patients in the passive arm on average are detected later and with more progressed disease than patients in the active arm. To the extent that this potential selection problem affect long term outcomes, we believe that the model still holds true as transition probabilities are informed by real study data.

## Conclusion

Our results shows that incentive-driven active case-finding in the private sector is cost-effective and should be added on to the routine passive case-finding in this setting if resources are available.

## Additional file

Additional file 1: **Table S1.** Ingredient Costing. Ingredient cost used to calculate each cost parameter in active and passive case finding arms. (DOCX 68 kb)

## Data Availability

The datasets used and/or analyzed during the current study are available from the corresponding author on reasonable request.

## Abbreviations

ABC: Activity based costing
ACF: Active case finding
DALYs: Disability-adjusted life years
DHHS: Department of Health and Human Services
GP: General practitioners
ICERs: Incremental cost-effectiveness ratios
NTP: National TB Control Programs
OHRP: Office of Human Research Protections
PCF: Passive case finding
TB: Tuberculosis
