# Correction to: The cost-effectiveness of incentive-based active case finding for tuberculosis (TB) control in the private sector Karachi, Pakistan

**DOI:** 10.1186/s12913-019-4673-1

**Published:** 2019-11-05

**Authors:** Hamidah Hussain, Amani Thomas Mori, Aamir J. Khan, Saira Khowaja, Jacob Creswell, Thorkild Tylleskar, Bjarne Robberstad

**Affiliations:** 1 Interactive Research and Development, Global, Singapore, Singapore; 2 Centre for International Health, Bergen, Norway; 3 Department of Global Public Health and Primary Care, |University of Bergen, Bergen, Norway; 4 Stop TB Partnership, Geneva, Switzerland; 5 Section for Ethics and Health Economics, Bergen, Norway

**Correction to: BMC Health Services Research (2019) 19:690**

**https://doi.org/10.1186/s12913-019-4444-z**

In the original publication of this article [1], an author’s name needs to be revised from Jacob Creswel to Jacob Creswell.

The original article has been corrected.

The authors apologize for any confusion this may have caused.
