# Supplementary material for: The cost-effectiveness of incentive-based active case finding for tuberculosis (TB) control in the private sector Karachi, Pakistan
Source: BMC Health Serv Res. 2019 Oct 12;19:690. doi: 10.1186/s12913-019-4444-z (PMC6790051; doi:10.1186/s12913-019-4444-z)
Supplement: Supplementary file 1 [file 12913_2019_4444_MOESM1_ESM.docx]

Table S1: Ingredient Costing

| Ingredient Costing | Active Case Finding | Passive Case Finding |
|---|---|---|
| | | |
| Cost of no TB Symptom at screening | **2.15** | **4.24** |
| Patient cost | 2 | 4 |
| screening program cost | 0.15 | 0.24 |
| Cost of no TB Test with Positive Symptom | **2.15** | **4.24** |
| Patient cost | 2 | 4 |
| screening cost | 0.15 | 0.24 |
| Cost of screening and negative sputum test | **7.15** | **11.24** |
| Patient cost | 4 | 8 |
| screening cost | 0.15 | 0.24 |
| Investigations | 3 | 3 |
| Cost of screening and no treatment if smear positive | **82** | **48** |
| Patient cost | 6 | 12 |
| Incentive | 20 | 0 |
| Program/ clinic Personnel | 28 | 18 |
| Screening cost | 0.15 | 0.24 |
| investigations | 11 | 11 |
| Supervision and Monitoring | 9 | 3 |
| Data cost | 8 | 4 |
| Cost of screening, tests and successful TB treatment | **223** | **171** |
| As in Table 2 | | |
